# Retraction: Association of self-leadership with acute stress responses, and acute stress disorders in Chinese medics during the COVID-19 epidemic: a cross-sectional study

**DOI:** 10.3389/fpsyt.2024.1524391

**Published:** 2024-11-13

**Authors:** Frontiers Editorial Office

The journal retracts the 10^th^ June 2022 article cited above.

Following publication, concerns were raised regarding the provenance of the patient data used in this study. During an investigation conducted in accordance with Frontiers’ policies, the authors confirmed the existence of a conflict over the ownership of the patient data between different research groups. Unauthorized use of data is a violation of Frontiers’ guidelines and those of the Committee on Publication Ethics. As such, this article is being retracted.

The retraction was approved by the Chief Executive Editor of Frontiers. The authors collaborated in the investigation and approved the retraction.

